# SIRT1 inhibition impairs non-homologous end joining DNA damage repair by increasing Ku70 acetylation in chronic myeloid leukemia cells

**DOI:** 10.18632/oncotarget.6455

**Published:** 2015-12-03

**Authors:** Wenjun Zhang, Haixia Wu, Meng Yang, Shiguang Ye, Liang Li, Hong Zhang, Jiong Hu, Xuguang Wang, Jun Xu, Aibin Liang

**Affiliations:** ^1^ Department of Hematology, Tongji Hospital, Tongji University School of Medicine, Shanghai, P.R. China; ^2^ Department of Clinical Pharmacology, Tongji Hospital, Tongji University School of Medicine, Shanghai, P.R. China; ^3^ Department of Hematology and Shanghai Institute of Hematology, Collaborative Innovation Center of Hematology, Ruijin Hospital, Shanghai Jiao Tong University School of Medicine, Shanghai, P.R. China; ^4^ Clinical Research Center, Affiliated Hospital of Guangdong Medical College, Zhanjiang, Guangdong, P.R. China; ^5^ East Hospital, Tongji University School of Medicine, Shanghai, P.R. China

**Keywords:** SIRT1, DNA damage repair, non-homologous end joining, Ku70

## Abstract

Most chemotherapeutic agents for leukemia are DNA damaging agents. However, DNA lesions can be repaired by activities of DNA repair systems. Increasing evidence have shown that enhanced DNA damage repair capacity contributes to chemotherapy resistance in leukemia cells. Thus, targeting DNA repair mechanisms is a promising strategy for novel leukemia treatment. SIRT1 expressions were downregulated by lentivirus-delivered SIRT1 shRNA in myeloid leukemia cells. SIRT1 mRNA and protein levels were analyzed by real-time PCR and Western blot, respectively. Flow cytometry was carried out to analyze cell cycle progression, apoptosis and DNA damage repair efficiency. DNA damage levels were assessed by alkaline comet assay, and H_2_AX phosphorylation was analyzed by immunoblotting and immunofluorescence. A mouse leukemia model was established by transplanting lentivirus-infected K562 cells containing SIRT1 shRNA into sublethally irradiated NOD/SCID mice, and tumorigenesis was evaluated by detecting tumor weights and mice survival. SIRT1 expressions were upregulated in myeloid leukemic patients. Downregulation of SIRT1 by RNAi promoted etoposide-induced DNA damage in myeloid leukemia cells accompanied by reduced NHEJ activity, and increased Ku70 acetylation. Furthermore, SIRT1 knockdown resulted in cell cycle arrest, induction of apoptosis and reduction of K562 cell proliferation accompanied by enhanced p53 and FOXO1 acetylation in K562 cells after etoposide treatment. Importantly, SIRT1 downregulation reduced the tumorigenesis ability of K562 cells in mouse xenografts following chemotherapy treatment. These results revealed that SIRT1 promotes the NHEJ repair pathway by deacetylating Ku70 in K562 cells, suggesting that SIRT1 is a novel therapeutic target for treating myeloid leukemia.

## INTRODUCTION

DNA damage-based chemotherapy is currently the first choice for treating leukemia. DNA damage leads to cell cycle arrest and cell death. However, the effect of DNA-damaging drugs can be reduced by enhanced activities of DNA repair pathways, which is one of the main mechanisms of anti-cancer drug resistance. Accumulating evidences have shown that DNA repair pathway modulation can sensitize a number of cancers to DNA damage-based cancer therapies [1]. Therefore, targeting the DNA repair system is a promising strategy for the development of novel leukemia treatments.

Different types of DNA damage are repaired by distinct repair systems such as base excision repair (BER), nucleotide excision repair (NER), mismatch repair (MMR), homologous recombination (HR) and non-homologous end joining (NHEJ) [2]. DNA-damaging drugs in leukemia therapy could cause several DNA lesions including point mutation, insertion, translocation, single-strand breaks (SSBs) and double-strand breaks (DSBs); among which DSBs are lethal if not repaired [3]. In eukaryotes, DSBs are mainly repaired by the NHEJ pathway; which include end-binding and end-processing proteins Ku70, Ku80, DNA-PKcs and Artemis, as well as ligation complexes XRCC4, LigIV and Cerrunos[4, 5]. It is well documented that enhanced NHEJ contributes to chemotherapy resistance in leukemia [6], suggesting that pharmacologically inhibiting the NHEJ repair pathway may sensitize leukemia cells to DNA-damage drugs.

SIRT1 is a mammalian NAD^+^ dependent protein deacetylase [7] that regulates longevity and a variety of physiological stress responses [8, 9] by deacetylating histones and non-histone proteins [10]. Through deacetylation, SIRT1 controls the activity of several DNA damage repair proteins including Ku70 [11], Nijmegen breakage syndrome protein (NBS1) [12], Werner syndrome protein (WRN) [13], and xeroderma pigmentosum C protein (XPC) [14]. It is widely accepted that epigenetic modifications of DNA repair machineries for facilitating DNA damage repair is one important function of SIRT1 in regulating cell physiology [15, 16].

Previous studies have shown that SIRT1 promotes acquisition of genetic mutations for drug resistance[17] and leukemogenesis[18] in CML (chronic myeloid leukemia). Here we investigated the higher expression of SIRT1 not only in CML but also acute myeloid leukemia (AML) cells and assessed the activity of NHEJ repair by downregulating SIRT1 in these myeloid leukemia cells. Our results have shown that silencing of SIRT1 reduced the efficiency of NHEJ repair and sensitized these myeloid leukemia cells to etoposide.

## RESULTS

### SIRT1 expressions are upregulated in leukemic patients

**Figure 1:**
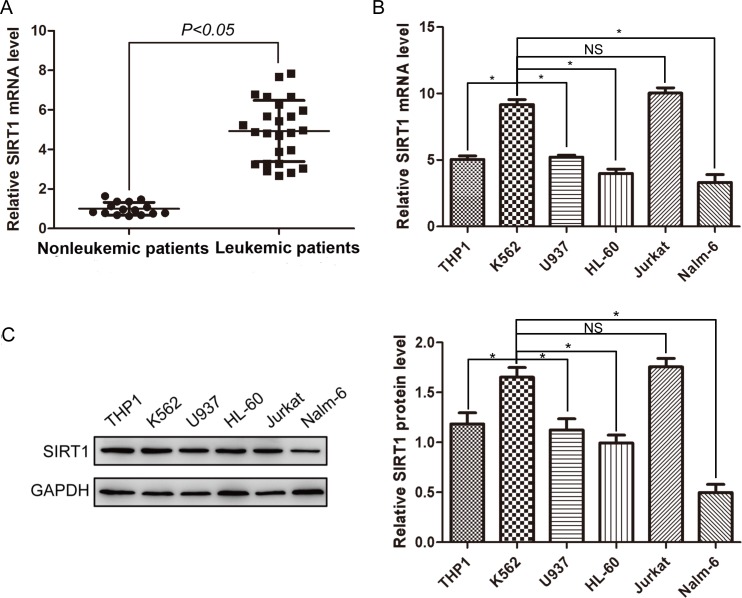
Expression of SIRT1 in MNCs from leukemic patients and leukemia cell lines **A.** Relative expression of SIRT1 in MNCs from 15 non-leukemic and 25 leukemic patients. Relative SIRT1 mRNA levels normalized to β-actin were analyzed by Real-time PCR. **B.** Relative expression of SIRT1 in six leukemia cell lines. Relative SIRT1 mRNA levels normalized to β-actin were analyzed by Real-time PCR. **C.** Protein expression of SIRT1 in six leukemia cell lines with β-actin as an internal control. *P < 0.05, NS indicated no significance.

To assess the expression status of SIRT1 in leukemia, we determined the mRNA levels of SIRT1 in mononuclear cells (MNCs) from 25 leukemic patients and 15 non-leukemic patients by real-time PCR. Details for patients are shown in Table 1. SIRT1 mRNA level in MNCs of leukemic patients was approximately 4.93 ± 1.55 times of non-leukemic patients (Figure 1A). This result indicates that SIRT1 expressions were significantly elevated in leukemia cells.

**Table 1:** Baseline characteristics of the patients

Variables	Leukemic Patients (*n* = 25)	Non-leukemia Patients (*n* = 15)
Age		
<60	16	10
>=60	9	5
Sex		
Female	14	9
Male	11	6
Classification of Diseases		
CML	8	-
AML	17	-
Anemia	-	9
ITP	-	6

We next determined the mRNA and protein levels of SIRT1 in several leukemia cell lines by real-time PCR and Western blot analysis, respectively. K562 cells demonstrated relatively higher levels of SIRT1 mRNA than other leukemia cell lines (Figure 1B). Similarly, relatively higher levels of SIRT1 proteins were observed in K562 cells, compared to other leukemia cell lines (Figure 1C).

### ShRNA-mediated downregulation of SIRT1 enhances etoposide-induced DNA damage in leukemia cells

**Figure 2:**
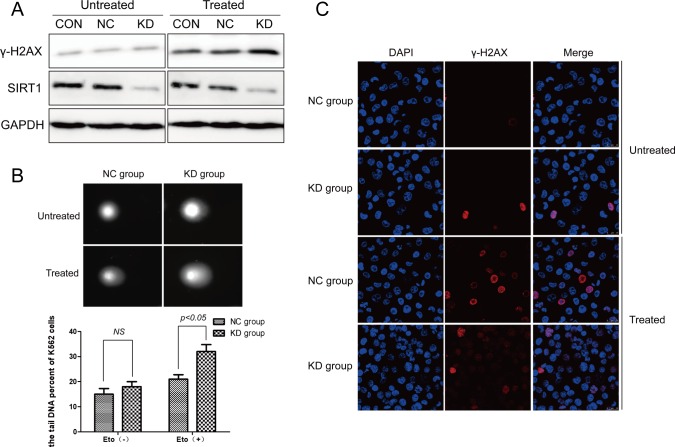
DNA damage was enhanced following SIRT1 knockdown in response to etoposide treatment **A.** K562 cells were infected with lentivirus carrying SIRT1 or control shRNA, and treated with or without etoposide. Total proteins were extracted for Western blotting of SIRT1, γ-H2AX and GAPDH. SIRT1 proteins decreased by 85.14% (*P* < 0.05) with SIRT1 shRNA compared with the CON group. No significant difference in SIRT1 protein expressions were observed between the CON and NC groups. **B.** Alkaline comet assay was performed to assess DNA damage after 20 μM of etoposide treatment for four hours in NC and SIRT1 knockdown (KD) cells. **C.** Representative immunofluorescence staining for γ-H2AX (red) and DNA (blue) in NC and SIRT1 knockdown (KD) cells with or without 20 μM of etoposide treatment for four hours.

To investigate the potential role of SIRT1 in DNA damage response in leukemia cells, K562 cells were infected with lentivirus expressing shRNA targeting SIRT1 (shSIRT1-KD) or negative control (shRNA-NC). Infection of shSIRT1-KD drastically reduced SIRT1 protein levels in K562 cells (Figure 2A). We then performed comet assay and recorded different comet parameters using Comet CASP, and used olive tail moment (OTM) to describe the extent of DNA damage. Silencing of SIRT1 apparently, but not significantly, increased OTM values in K562 cells under normal growth conditions. However, a significant increase (*P* < 0.05) in DNA strand breaks, as indicated by an increase in OTM, was observed following SIRT1 knockdown (32.09 ± 3.13) after etoposide treatment in K562 cells, compared to the NC group (21.76 ± 1.96) (Figure 2B). Consistent with comet assay results, Western blot analyses revealed that treatment of 20 μM of etoposide resulted in increased levels of γ-H2AX, a marker of DSBs, in K562 cells infected with shSIRT1-KD, compared with that of cells infected with shRNA-NC (Figure 2A). Further immunofluorescence staining demonstrated an increased number of γ-H2AX foci in K562 cells infected with shSIRT1-KD, compared with that of cells infected with shRNA-NC following etoposide treatment (*P* < 0.05, Figure 2C). These results clearly demonstrate that the silencing of SIRT1 lead to enhanced DNA damage in response to etoposide treatment in K562 cells. Interestingly, downregulation of SIRT1 also resulted in increased levels of γ-H2AX following etoposide treatment in THP-1 and U937 cells (Supplementary Figure S1).

### Inhibition of SIRT1 reduces the efficiency of NHEJ but not HR

**Figure 3:**
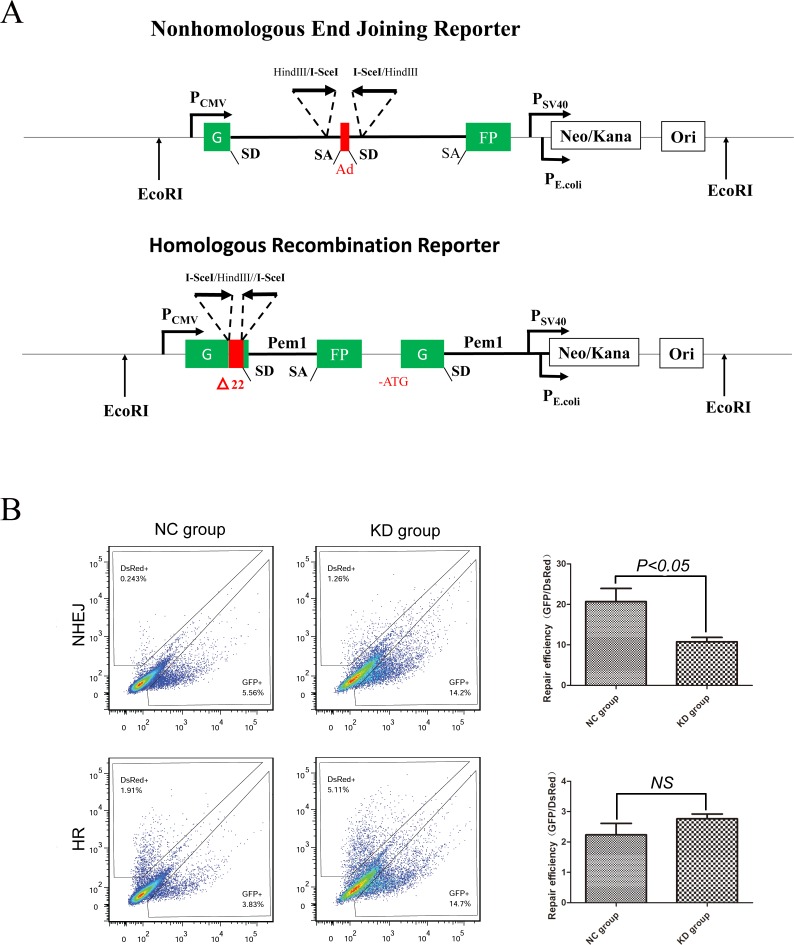
Silencing of SIRT1 reduced NHEJ efficiency, but not HR **A.** Reporter constructs for analysis of NHEJ and HR repair. **B.** Flow cytometry was carried out to analyze HR and NHEJ repair efficiency after SIRT1 knockdown using reporter constructs digested in vitro with I-SceI endonuclease, and transfected into K562 cells as linear DNA. DS-Red was used for transfection control. Repair rate was normalized to DS-Red.

To analyze the efficiency of DNA damage repair in a quantitative manner, we used fluorescent reporter constructs in which a functional GFP gene is reconstituted following an HR or NHEJ event (Figure 3A). K562 cells infected with shSIRT1-KD or shRNA-NC were transfected with plasmids containing green fluorescent protein-based reporter constructs by electrotransfer; which allowed for the separate analysis of HR and NHEJ. Results revealed that SIRT1 knockdown by shSIRT1 reduced the efficiency of NHEJ repair to 50% compared with the shRNA-NC group (*P* < 0.05), but did not significantly reduce the efficiency of the HR pathway (*P* > 0.05, Figure 3B). This result indicates that SIRT1 was required for NHEJ in K562 cells. The similar results were also observed in THP-1 and U937 cells (Supplementary Figure S2).

### SIRT1 knockdown induces cell cycle arrest and apoptosis, and reduces K562 cell proliferation

**Figure 4:**
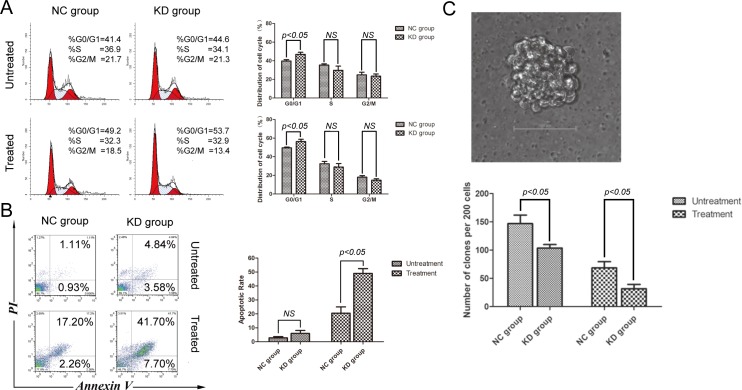
SIRT1 knockdown induced cell cycle arrest and apoptosis, while reducing the clonogenic capacity of K562 cells **A.** Effects of SIRT1 knockdown on cell cycle distribution of K562 cells. Representative flow histograms depicting cell cycle distribution of K562 cells with or without 20 μM of etoposide treatment for four hours are shown. **B.** Effects of SIRT1 knockdown on K562 cell apoptosis. Three days after infection of lentivirus containing negative control shRNA (NC) or shSIRT1, 20μM of etoposide was added for four hours; and apoptosis was analyzed 48 hours later by flow cytometry. **C.** Effects of SIRT1 knockdown on K562 cell colony formation. Two hundred NC and SIRT1-KD cells per plate with or without 20μM of etoposide treatment for four hours were seeded in standard 2-layer soft agar in triplicate. Colonies were scored after seven days. SIRT1 knockdown significantly inhibited soft agar colony formation of K562 cells. Error bars represent SD from three experiments.

Silencing of SIRT1 impaired NHEJ and enhanced etoposide-induced DNA damage. We examined the cell cycle distribution of K562 cells in the two groups following treatment with etoposide. As shown in Figure 4A, the proportion of G_0_/G_1_ phase cells in the shSIRT1-KD group was 46.87±2.20% *versus* 39.70±1.48% in the shRNA-NC group (*P* < 0.05). After etoposide treatment, proportions of G_0_/G_1_ phase cells were 56.30±2.39% in the KD group and 49.53±0.85% in the NC group, respectively (*P* < 0.05). These results indicate that silencing of SIRT1 resulted in cell cycle arrest at G0/G1 phase under normal growth conditions and in response to etoposide treatment.

Another key function of DNA damage checkpoints is to induce apoptosis in order to eliminate cells with irreparable DNA damages. We further examined the apoptotic rate in both groups by annexin V staining coupled with flow cytometry. As shown in Figure 4B, apoptotic rate in the shSIRT1-KD group was higher than in the shRNA-NC group. Following treatment with 20 μM of etoposide for four hours, a significant increase in apoptotic rate was observed in the shSIRT1-KD group compared with the shRNA-NC group after 48 hours (*P* < 0.05); demonstrating that SIRT1 knockdown enhanced cell apoptosis in response to etoposide treatment in K562 cells. Another set of the same experiment was performed, and SIRT1 knockdown also enhanced cell apoptosis in response to etoposide treatment in THP-1 and U937 cells (Supplementary Figure S3).

To explore the role of SIRT1 in regulating K562 cell proliferation, we performed a colony formation assay with soft agar. Compared with negative control (NC) cells, SIRT1 knockdown reduced colony formation in K562 cells by 29% without any drug treatment (*P <* 0.05) and by 54% following four hours of etoposide treatment (*P <* 0.05) (Figure 4C); indicating that silencing of SIRT1 results in loss of cell viability under normal growth conditions and in response to etoposide treatment.

### SIRT1 downregulation reduces the tumorigenesis ability of K562 cells

**Figure 5:**
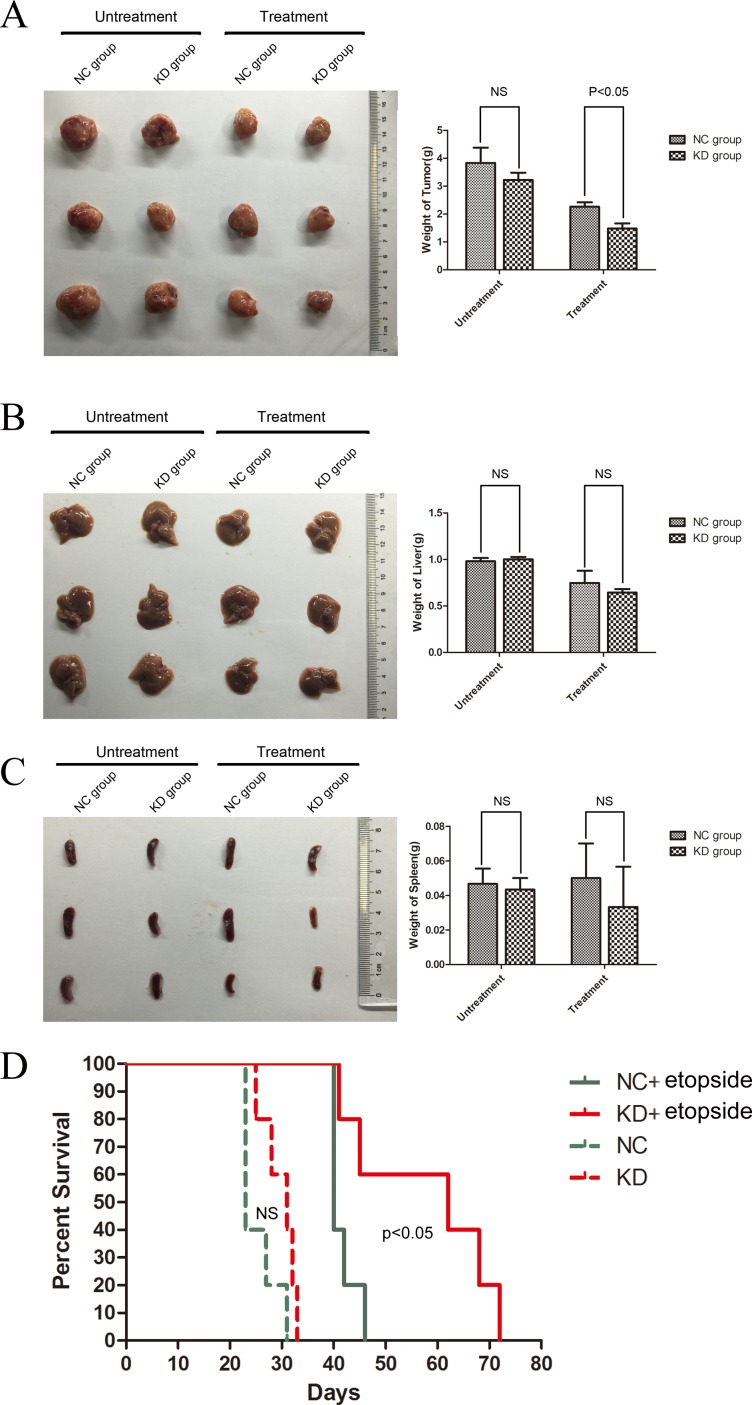
Downregulation of SIRT1 decreased the tumorigenesis of K562 cells *in vivo* The weights of tumor **A.**, liver **B.** and spleen **C.** four weeks after transplantation. **D.** Survival curves of mice receiving xenografted K562 cells. The shSIRT1-KD group had a longer life-span than the shRNA-NC group after etopside treatment(*p* < 0.05).

To extend our observations *in vivo*, we transplanted ShSIRT1-KD and shRNA-NC K562 cells with or without etoposide treatment *via* subcutaneous injection into sublethally irradiated (250 cGy) NOD/SCID mice, and evaluated l tumorigenesis by detecting tumor weights and mice survival. As shown in Figure 5A, four weeks after transplantation, although there is no significant difference in tumor weights between the shSIRT1-KD group and shRNA-NC group without etopside treatment, the shSIRT1-KD group showed marked retardation in tumor growth (1.48±0.19g), compared with shRNA-NC group(2.26±0.16g, *p* < 0.05) after etoposide treatment. However, four weeks after engraftment, the weights of livers and spleens in the shSIRT1-KD group were similar to the control group (Figure 5B and 5C). In addition, the group engrafted with shSIRT1-KD K562 cells had a longer life-span than the shRNA-NC group after etopside treatment (Figure 5D). These results revealed that SIRT1 inhibition effectively reduced the tumorigenesis ability of K562 cells *in vivo*.

### SIRT1 inhibition enhances Ku70, p53 and FOXO1 acetylation in leukemia cells after etoposide treatment

**Figure 6:**
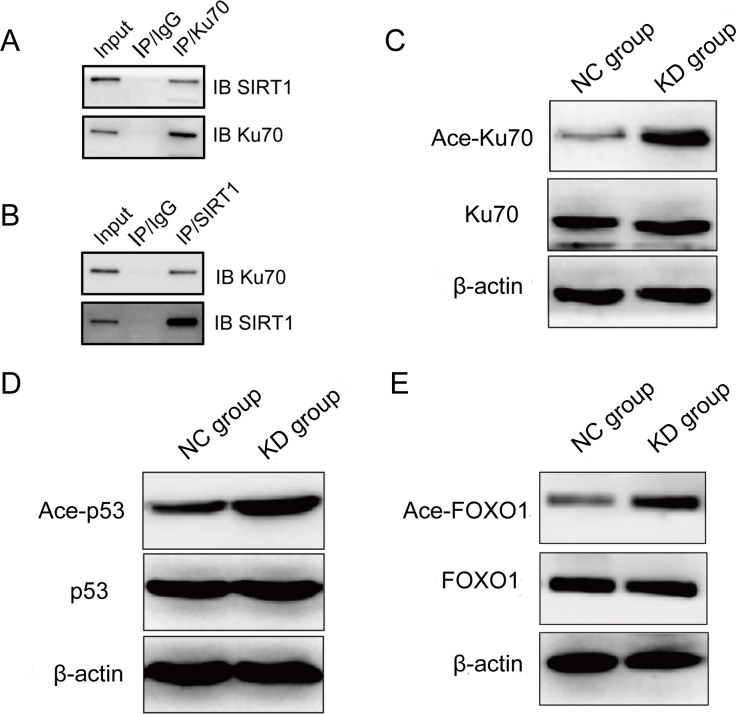
SIRT1 physically interacted with Ku70 and deacetylated Ku70, p53 and FOXO1 in leukemia cells after etoposide treatment Cell lystes were immunoprecipitated with anti-Ku70 **A.**, anti-SIRT1 antibody **B.** or control IgG antibody; and then, reciprocally probed with anti-SIRT1 **A.** or anti-Ku70 antibody **B.**, respectively. **C.** Acetylation of Ku70 after SIRT1 knockdown in K562 cells treated with 20 μM of etoposide for four hours. Ku70 was immunoprecipitated from the total cell lysate of NC or shSIRT1 knockdown cells by Ku70 antibody; And then, western blots were probed with anti-acetylated lysine antibody. Representative Western blot analysis of p53 **D.** and FOXO1 **E.** acetylation change in K562 cells with etoposide treatment after SIRT1 knockdown.

To explore the molecular mechanism of SIRT1 in regulating the NHEJ repair pathway in K562 cells, we assessed the interaction between SIRT1 and Ku70, a core component in the NHEJ repair pathway, in the lysate of K562 cells after etoposide treatment by immunoprecipitation. After etoposide treatment, cells were lysed and subsequently immuno-precipitated with anti-Ku70 antibody. Resulting immune complexes were analyzed by immunoblotting with anti-SIRT1 antibodies. As shown in Figure 6A, immunoprecipitation of Ku70 from lysates of K562 cells resulted in coimmunoprecipitation of SIRT1. This interaction was reciprocally further confirmed by immunoprecipitating the lysate with anit-SIRT1 antibodies, and subsequently probing the blotted precipitate with Ku70 antibodies (Figure 6B). Importantly, after SIRT1 knockdown, Ku70 acetylation levels significantly increased (Figure 6C). These results suggest that SIRT1 physically forms a complex and subsequently deacetylases Ku70 in K562 cells. Thus, it is possible that SIRT1 knockdown reduces NHEJ repair pathway efficiency by increasing Ku70 acetylation levels, which is an inactive form of Ku70.

In response to DNA damage, p53 and FOXO1 play an important role in the induction of cell cycle arrest, apoptosis and proliferation inhibition [19, 20]. Moreover, deacetylation of p53 plays an important role in SIRT1-mediated cell survival [21], and FOXO1 is a SIRT1 substrate [22]. The induction of cell cycle arrest, apoptosis and proliferation inhibition following SIRT1 knockdown suggests that SIRT1 may deacetylase p53 and/or FOXO1. To test this hypothesis, we detected p53 and FOXO1 acetylation levels by immunoblotting. SIRT1 knockdown did not change the total level of p53, but increased the level of p53 acetylation after etoposide treatment (Figure 6D). In addition, SIRT1 knockdown increased FOXO1 acetylation without affecting the total FOXO1 protein expression (Figure 6E).

## DISCUSSION

**Figure 7:**
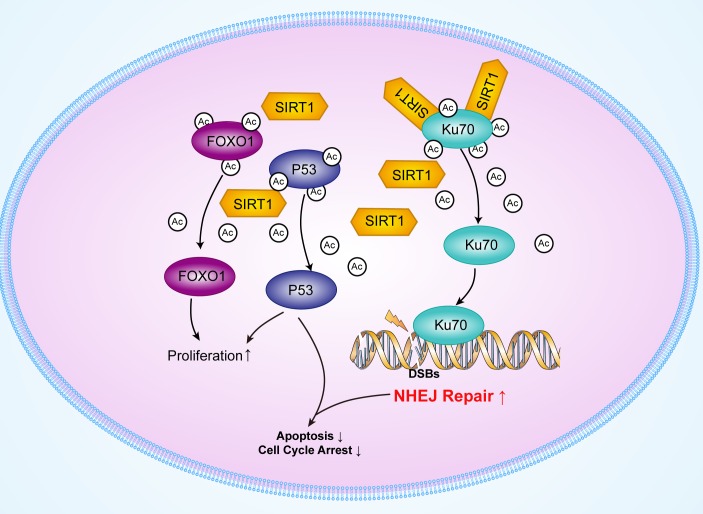
Schematic model of the upregulation of Ku70, p53 and FOXO1 deacetylation by SIRT1

In this present study, we demonstrated that SIRT1 expressions were upregulated in myeloid leukemic patients. Moreover, SIRT1 downregulation by RNAi promoted etoposide-induced DNA damage in chronic myeloid leukemia cells accompanied with reduced NHEJ activity, but increased Ku70 acetylation. Furthermore, SIRT1 knockdown resulted in cell cycle arrest, induction of apoptosis, and reduction of cell proliferation in K562 cells accompanied by enhanced p53 and FOXO1 acetylation in myeloid leukemia cells after etoposide treatment. Importantly, SIRT1 downregulation reduced the tumorigenesis ability of K562 cells in mouse xenografts following chemotherapy treatment. Additionally, silencing of SIRT1 also impaired the DNA damage repair ability with reduced NHEJ activity, leading to upregulation of cell apoptosis in THP-1 and U937, which are acute myeloid leukemia cell lines. Our results suggest that SIRT1 upregulation may promote the progression of leukemia by enhancing NHEJ and suppressing p53 and FOXO1 activity, and that targeting SIRT1 is a novel anticancer therapeutic strategy for treating leukemia (Figure 7).

In mammalian cells, SIRT1 is known to enhance DNA damage repair [23]. Consistently, our results revealed that SIRT1 was upregulated in myeloid leukemic cells. SIRT1 inhibition reduced NHEJ repair efficiency in the K562 chronic leukemia cell line probably *via* Ku70 inactivation, which is a key component in the NHEJ pathway, indicating an important role for SIRT1 in the DNA damage response of myeloid leukemia.

Etoposide, which is a topoisomerase II inhibitor [24], stabilizes the cleavage complex; leading to topoisomerase II mediated chromosome DNA breakage [25]. In this study, cells were treated with etoposide for four hours at a concentration of 20 μM [26]; which could induce DNA damage in myeloid leukemia cells. All the analyses were performed 48 hours later, which provided enough time for DNA damage repair in myeloid leukemia cells. The remaining lesions in both shSIRT1-KD and shRNA-NC cells after treatment with etoposide were investigated by two methods. One method was the comet assay, which was used to detect DNA damage caused by DSBs, SSBs, alkali labile sites, oxidative base damage, and DNA cross-linking with DNA or proteins [27]. Repair capacities of all above lesions were compared by the parameter OTM. Our results revealed that the reduction of endogenous SIRT1 expressions by SIRT1 shRNA in K562 cells resulted in an increase of OTM. The other method was immunofluorescence and immunoblotting analysis, which was used to detect γ-H2AX. Histone H2AX phosphorylation on serine-139 (γ-H2AX) is a sensitive marker for DNA DSBs, and the number of foci has been found to correlate closely with the number of DSBs [28]. We found that SIRT1 knockdown significantly increased the number of γ-H2AX foci after etoposide treatment. These results indicate that SIRT1 knockdown decreased the repair capacity of DNA lesions, especially DSBs, which was induced by etoposide in K562 cells.

Furthermore, our results demonstrated that SIRT1 knockdown significantly decreased the efficiency of NHEJ, but not HR. NHEJ is an evolutionarily conserved and predominant DSBs repair pathway in mammalian cells [29]. Ku70 is a protein encoded by the XRCC6 gene in humans, and a core component of the NHEJ repair pathway [5]. Together with Ku80, Ku70 forms the Ku heterodimer that binds to DNA DSBs ends, and is essential for DNA repair by the NHEJ pathway. A previous study has shown that SIRT1 enhanced DNA repair activity, physically formed complexes with repair protein Ku70, and subsequently deacetylated Ku70 in 293 cells [11]; indicating that SIRT1 could enhance DNA repair capacity through the deacetylation of Ku70. In this present study, SIRT1 knockdown increased Ku70 acetylation levels (an inactive form of Ku70) after etoposide treatment in K562 cells. Our results suggest that SIRT1 may stimulate the NHEJ repair pathway by deacetylating Ku70 in myeloid leukemia cells.

While appropriate DNA damage repair restores cellular functions, cells with excessive damage undergo transient cell-cycle arrest or apoptosis. In this study, SIRT1 knockdown led to cell cycle arrest in the G_0_/G_1_ phase and increased K562 cell apoptosis in response to etoposide treatment. Mechanistically, we found that SIRT1 knockdown led to the increase of p53 acetylation (an active form of p53). Activation of p53 leads to cell cycle arrest in the G_0_/G_1_ phase and promotes apoptosis. In addition, SIRT1 knockdown inhibited K562 cell clonal proliferation. It was reported that SIRT1-mediated FOXO1 deacetylation plays a key role in regulating diverse cellular processes such as differentiation and proliferation in cancer cells [30, 31]. Consistently, we have shown that SIRT1 knockdown increased FOXO1 acetylation levels, which may partially be responsible for the inhibition of K562 cell clonal proliferation.

## CONCLUSIONS

Our study suggests that SIRT1 deacetylase enhances NHEJ DNA damage repair efficiency in myeloid leukemia cells, and that targeting SIRT1 may provide a novel strategy for improving leukemia treatment and for overcoming DNA damage based chemotherapy resistance.

## MATERIALS AND METHODS

### Cell culture and drug treatment

Leukemia cell lines HL-60, K562, U937, THP-1, Jurkat, Nalm-6, as well as lentivirus packaging cell line-293T cells, were purchased from the Shanghai Institute for Biological Sciences, Chinese Academy of Sciences (Shanghai, China). The 293T cells were grown in Dulbecco's modified Eagle's medium (DMEM) containing 10% fetal bovine serum (FBS). Leukemia cells were maintained in RPMI-1640 medium supplemented with 10% FBS. Cells were incubated at 37°C with 5% CO_2_ humidity. Cells with logarithmic growth phase were used for further experiments. Cells were seeded in 24-well plates at 1 × 10^5^ cells/ml, treated with etoposide (Sigma, St. Louis, MO) at a concentration of 20 μM, and incubated for four hours. Then, cells were washed in PBS for three times and cultured in RPMI-1640 medium supplemented with 10% FBS until further analysis.

### Human mononuclear cells (MNCs)

Human mononuclear cells (MNCs) were separated from bone marrow drawn from 25 leukemic patients and 15 non-leukemic patients. All participants were informed on the purpose of the tests and written informed consent was obtained from each participant. This study was approved by the Ethical Committee of Tongji Hospital, Tongji University School of Medicine. After obtaining informed consent, 3 ml of bone marrow from each individual was collected in tubes containing EDTA as the anti-coagulant agent; then, MNCs were separated with Ficoll, and cultured under regular conditions or stored at −80°C for further use. The proportion of leukemia cells in all samples was > 90% and trypan blue excluding fraction was > 98%.

### RNA interference targeting SIRT1

The anti-SIRT1 ShRNA sequence-GAAGTGCCTCAGATATTAA was used for SIRT1 downregulation (shSIRT1-KD). The siRNA sequence- TTCTCCGAACGTGTCACGT unrelated to SIRT1 was synthesized and served as negative control (shRNA-NC) [18]. ShRNAs were inserted into pLVX-shRNA vectors containing a puromycin (shRNA1) or ZsGreen (shRNA2) expression cassette, yielding recombinant vectors. Recombinant vectors together with two lentiviral packaging plasmids (at a 4:3:2 ratio) were transfected into 293T cells using Lipofectamine 2000 (Invitrogen). Serial dilution method and flow cytometry were performed to determine the lentivirus titer. The lentivirus of shSIRT1-KD and shRNA-NC was used to infect K562 cells at appropriate titers, and sh-KD-K562 and sh-NC-K562 were named as KD group and NC group, respectively. In addition, untreated K562 cells were used as controls (CON group). The proportion of GFP-positive cells was observed under a fluorescence microscope 72 hours later. Cells were used for subsequent experiments when the proportion was > 90%. These cells were harvested 120 hours after transfection; and real-time PCR and Western blot analysis were performed to determine the efficiency of RNA interference on mRNA and protein expression levels. The experiment was repeated three times.

### RNA extraction and real-time PCR

RNA was extracted with MiniBEST Universal RNA Extraction Kit (TaKaRa) according to manufacturer's protocol, and was immediately converted to cDNA with PrimeScript™ II 1st Strand cDNA Synthesis Kit (TaKaRa). Fluorescence quantitative PCR instrument (Applied Biosystems 7500 Fast Real-Time PCR Systems, Life Technologies, Carslbad, CA, USA) and a SYBR^®^ Premix Ex Taq™ kit (TaKaRa) were used to detect target gene expressions, and β-actin was used as an internal reference. Sequences of primers were as follows:

SIRT1 forward: CAGGTCAAGGGATGGTATT TATGC;
reverse: TTCAATATCAAACATCGCTTGAGG;
β-actin forward: GAACGGTGAAGGTGACAGCAG;
reverse: GTGGACTTGGGAGAGGACTGG.

The 2^−ΔΔCT^ method was employed to determine the relative expression of target genes normalized to β-actin, and experiments were repeated in triplicate.

### Protein extraction, western blotting and immunoprecipitation

Cells were harvested and lysed in lysis buffer for protein extraction followed by the determination of protein concentration, and the supernatant was used for Western blotting. Proteins were separated by SDS-PAGE and transferred onto polyvinylidene fluoride (PVDF) membranes. The membranes were incubated with primary antibodies (Rabbit polyclonal anti-human SIRT1 [Epitomics], 1:1000; rabbit anti-human Ku70 [Epitomics], 1:1000; rabbit polyclonal anti-acetylated p53 [Cell Signaling Technology], 1:1000; rabbit polyclonal anti-acetylated FOXO1 [Santa Cruz Biotechnology], 1:1000; β-actin [Cell Signaling Technology], 1:1000) followed by incubation with secondary antibodies conjugated to horseradish peroxidase (HRP-donkey-anti-rabbit). Signal development was performed with an ECL kit. The experiment was performed three times. To analyze Ku70 acetylation, Ku70 was pulled down from total cell lysate with conjugated anti-Ku70-agarose beads (Santa Cruz Biotechnology), followed by acetylation detection with rabbit anti-acetyl lysine antibody (Cell Signaling Technology). Moreover, in order to analyze the direct interaction between SIRT1 and Ku70, SIRT1 or Ku70 was pulled down from total cell lysate with conjugated anti-SIRT1 or Ku70-agarose beads (Santa Cruz Biotechnology), followed by Ku70 or SIRT1 detection with rabbit anti-Ku70 or SIRT1 antibody (Cell Signaling Technology).

### Immunofluorescence analysis

Cells grown on coverslips were fixed in 4% paraformaldehyde, permeabilized with PBS containing 0.1% Triton X-100 (Sigma), and blocked in 3% normal donkey serum in PBS for 30 minutes at room temperature. Rabbit anti-human γH_2_AX (Abcam; 1:1000) antibody was diluted in 3% normal donkey serum in PBS and applied at 4°C overnight. After rinsing with PBS three times and incubating for one hour with donkey anti-rabbit secondary antibody (CF543, Biotium; 1:1000), slides were washed three times in PBS and cell nuclei were stained with DAPI (Invitrogen) for 10 minutes at room temperature. Images were acquired on a Leica TCS SP2 confocal fluorescence microscope.

### Apoptosis and cell cycle analysis

Apoptosis assay was performed using a BD-Bioscience Annexin V-APC staining kit according to manufacturer's protocols. Cells were seeded in 24-well plates at a density of 1 × 10^5^ cells/ml and maintained in a medium containing etoposide (20 μM) or PBS for four hours. Then, cells were washed in PBS three times and incubated for 48 hours. Cells (0.5 × 10^5^) were collected, washed with PBS twice, and stained with APC-Annexin V and PI. Apoptotic cells (Annexin V positive, PI negative) were determined by flow cytometry.

For cell cycle analysis, cells were resuspended in 1 ml of PBS, added 4 ml of cold absolute ethanol, and fixed at −20°C for 15 minutes followed by centrifugation. The supernatant was removed and 5 ml of PBS were added to the sediment followed by incubation for 15 minutes. Cells were collected by centrifugation and stained with DNA staining solution (50 mg/L propidium iodide [PI] and 10 mg/ml of RNase A) in the dark for 30 minutes. Flow cytometry was performed to analyze cell cycle distribution within one hour.

### Soft agar colony formation assay

For clonogenic assay, a standard two-layer soft agar culture was performed with a 0.6% agarose bottom layer and a 0.3% agarose top layer. Colonies were scored after seven days.

### Alkaline comet assay

At indicated time points after etoposide treatment, cells were harvested and washed with ice-cold PBS. Comet assay was performed by Comet Assay Kits (Cell Biolabs, Inc.) according to manufacturer's instructions. In brief, 5,000 cells were combined with OxiSelect™ comet agarose at 37°C, pipetted onto the OxiSelect™ comet slides, and placed at 4°C. Once solidified, the slides were immersed in pre-chilled lysis solution for one hour at 4°C or overnight. Then, lysis, slides were electrophoresed in alkaline electrophoresis buffer (PH > 13) at 300 mA for 30 minutes. Slides were then fixed in ethanol for 10 minutes and immediately stained with 30 μL of 1 x ethidium bromide staining solution prior to analysis. Comets were analyzed using a Nikon microscope fitted with a 20X objective and connected to a computer through a charge coupled device (CCD) camera to transport images to the software (Comet Assay Software Project, CASP 1.2.3 beta 1) for analysis. The final magnification was 400X. Two hundred cells per individual were randomly scored. The parameter used as metrics of DNA damage was OTM.

### DNA damage repair assays

Plasmids containing NHEJ, HR reporter cassettes and pDsRed-N1 as the internal controls were kindly provided by Dr Zhiyong Mao from the School of Life Science and Technology of Tongji University(Shanghai, China). Plasmids containing NHEJ or HR reporter cassettes were linearized by I-SceI restriction enzymes and purified using Qiagen Qiaex II purification kit (20021; Qiagen, Valencia, CA). Cells were transfected with 0.5 μg of NHEJ reporter construct or 2 μg of HR reporter construct, and 0.1 μg of pDsRed-N1 as internal control. Transfections were performed using an Amaxa Nucleofector (Walkersville, MD). Cells were analyzed by flow cytometry three days after transfection [32].

### Engraftment of K562 cells in immunodeficient mice

For the CML tumor xenograft assay, 2×10^7^ cells virally transduced with shSIRT1-KD or shRNA-NC (100μl injection volume containing 50% Becton Dickenson Matrigel; BD, San Jose, CA) with or without etopside treatment (20 μM of etoposide treatment for four hours) were inoculated subcutaneously into the right flank of NOD-SCID mice (South model animal center, China) conditioned by 250 cGy of irradiation. Tumors were measured with calipers and volumes were determined: V = (0.5)(LW^2^). Mice were euthanized when the tumor volume reached 1000mm^3^,and the survival curve was recorded to compare tumorigenesis ability between the NC group and KD group. Another set of the same experiment was performed to observe the histology of tumor, liver and spleen. Four weeks after transplantation, mice were euthanized, and the tumor, liver and spleen were obtained.

### Statistical analysis

Statistical analyses were performed with the SPSS version 19.0 software package (SPSS Inc, Chicago, IL) and GraphPad Prism Version 5.01 software for Windows (GraphPad Software, San Diego, CA USA). Two-tailed *t*-test analysis was used in all cases and *P* < 0.05 was considered statistically significance. Mice survival was calculated by the Kaplan-Meier method, and the log-rank test was applied to calculate the significance of differences between survival curves.

## SUPPLEMENTARY MATERIAL FIGURES


