# Supplementary material for: SIRT1 inhibition impairs non-homologous end joining DNA damage repair by increasing Ku70 acetylation in chronic myeloid leukemia cells
Source: Oncotarget. 2015 Dec 3;7(12):13538–50. doi: 10.18632/oncotarget.6455 (PMC4924659; doi:10.18632/oncotarget.6455)
Supplement: Supplementary file 1 [file oncotarget-07-13538-s001.pdf]

SIRT1 inhibition impairs non-homologous end joining DNA damage repair by increasing Ku70 acetylation in chronic myeloid leukemia cells

Supplementary Material

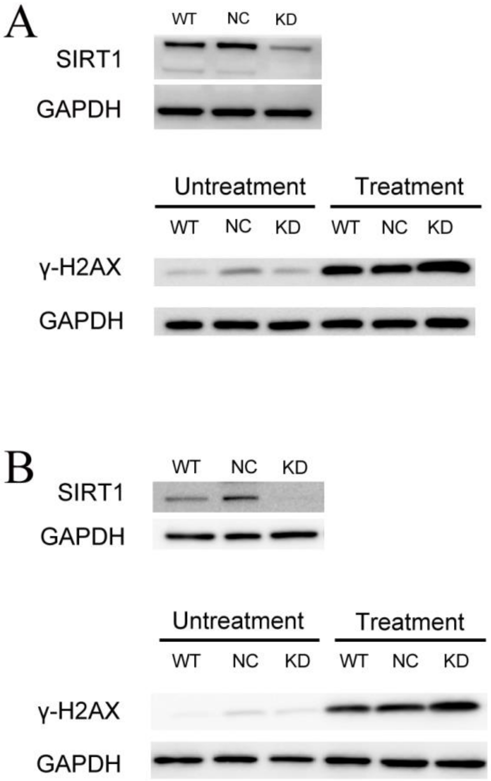

Figure S1: Inhibition of SIRT1 impairs DNA repair in THP-1(A) and U937(B) cells. Increased levels of γ-H2AX in THP-1 and U937 cells infected with shSIRT1-KD, compared with that of cells infected with shRNA-NC or Wild type(WT) cells following etoposide treatment.

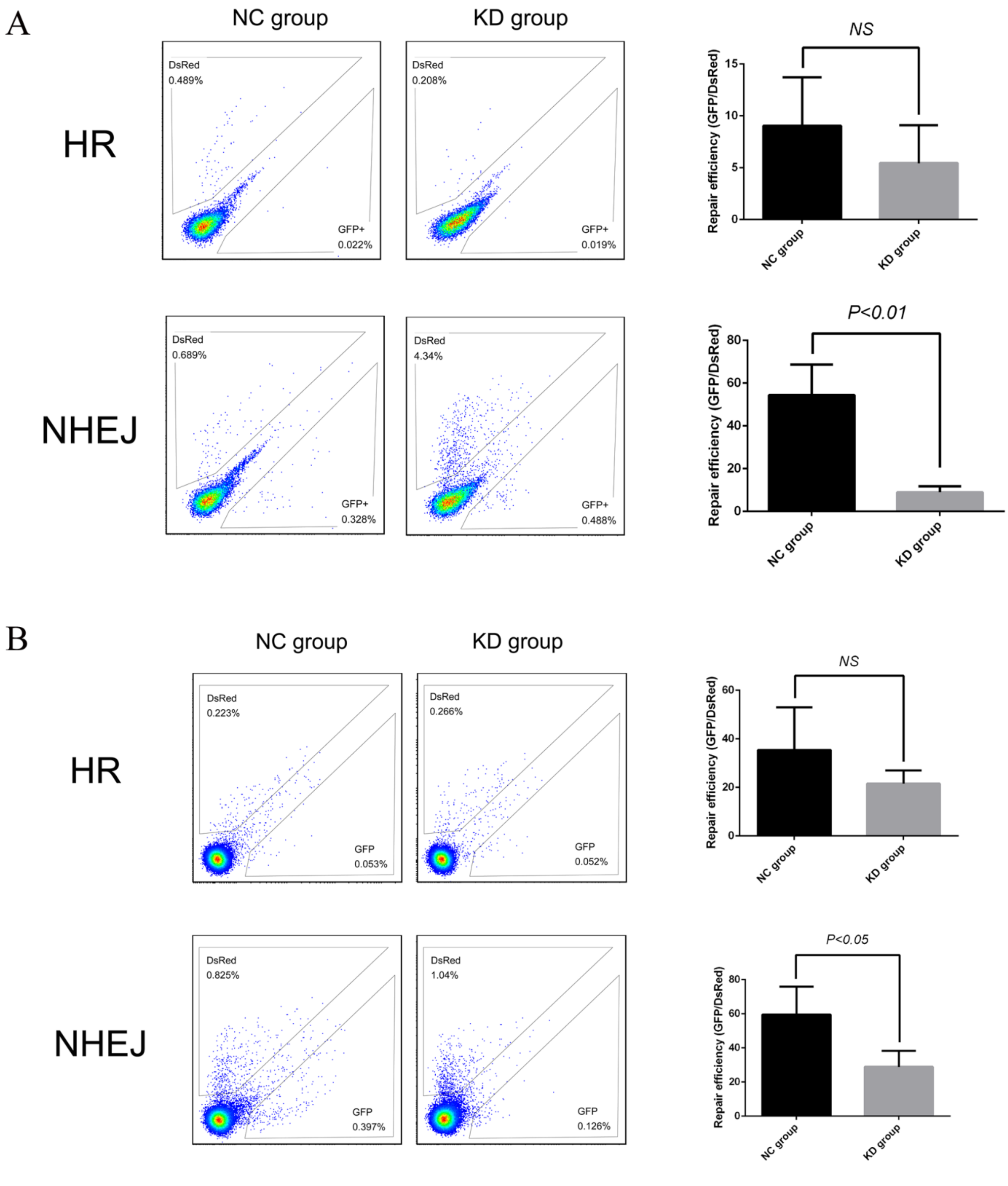

Figure S2: Analysis of HR and NHEJ in THP-1(A) and U937(B) cells. NHEJ but not HR is downregulated in THP-1 and U937 cells following silencing of SIRT1.

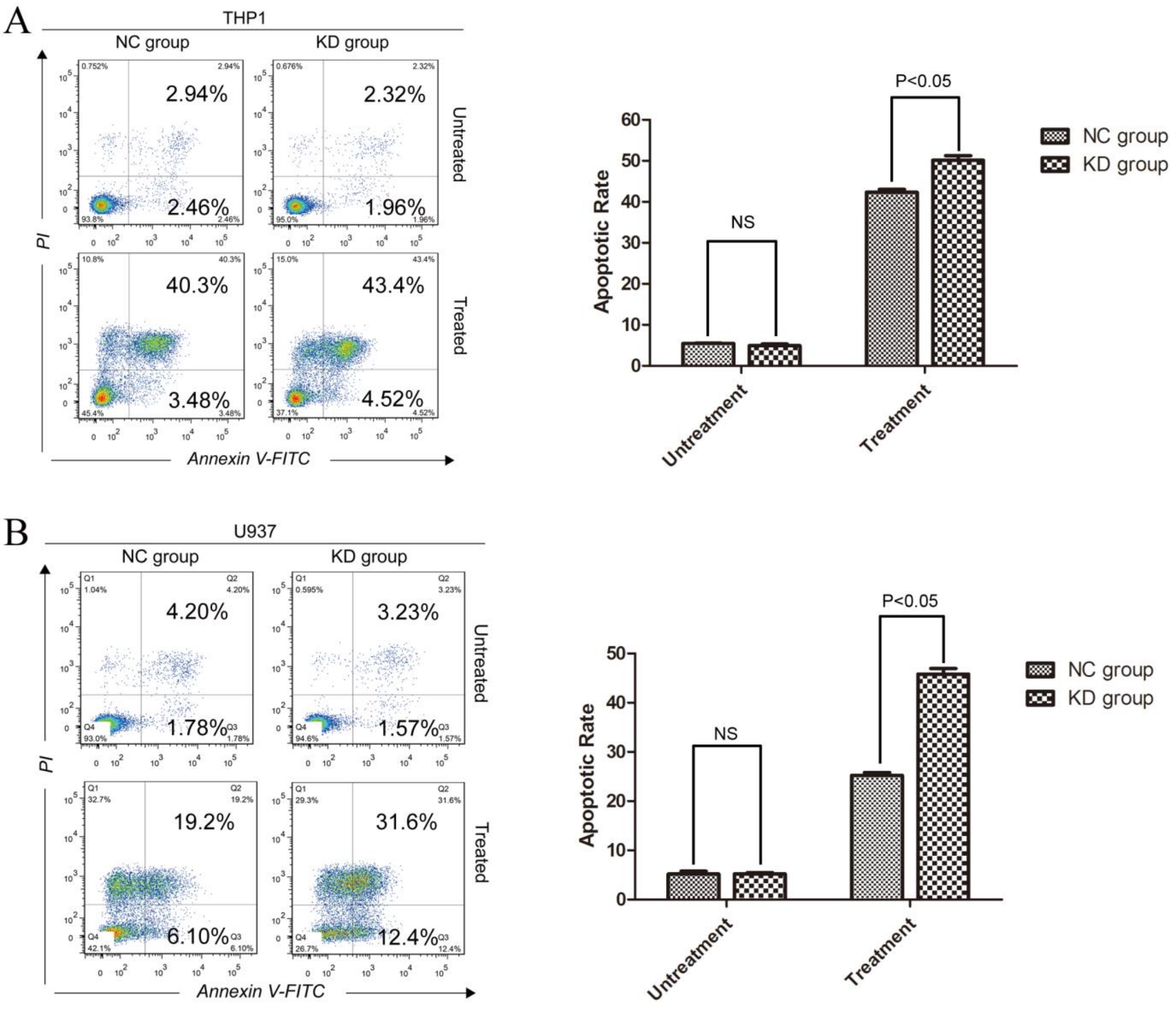

Figure S3: Effect of SIRT1 knockdown on survival of THP-1(A) and U937(B) cells. Silencing of SIRT1 induced cell apoptosis in THP-1 and U937 cells.
